# Completion of the swine genome will simplify the production of swine as a large animal biomedical model

**DOI:** 10.1186/1755-8794-5-55

**Published:** 2012-11-15

**Authors:** Eric M Walters, Eckhard Wolf, Jeffery J Whyte, Jiude Mao, Simone Renner, Hiroshi Nagashima, Eiji Kobayashi, Jianguo Zhao, Kevin D Wells, John K Critser, Lela K Riley, Randall S Prather

**Affiliations:** 1 National Swine Resource and Research Center, University of Missouri, 920 E. Campus Dr, Columbia, MO, 65211, USA; 2 Molecular Animal Breeding and Biotechnology, Department of Veterinary Sciences and Laboratory for Functional Genome Analysis, Feoder-Lynen-Strasse 250, Munich, 81377, Germany; 3 Center for Development of Advanced Technology, Jichi Medical University, 3311-1 Yakushiji, Shimotsuke-shi, Tochigi-ken, 329-0498, Japan; 4 Laboratory of Developmental Engineering, Meiji University, 1-1-1 Higashimita, Tama, Kawasaki, 214-8571, Japan

**Keywords:** Genomic, Pig, Biomedical model, Genetically engineered, Human diseases

## Abstract

**Background:**

Anatomic and physiological similarities to the human make swine an excellent large animal model for human health and disease.

**Methods:**

Cloning from a modified somatic cell, which can be determined in cells prior to making the animal, is the only method available for the production of targeted modifications in swine.

**Results:**

Since some strains of swine are similar in size to humans, technologies that have been developed for swine can be readily adapted to humans and vice versa. Here the importance of swine as a biomedical model, current technologies to produce genetically enhanced swine, current biomedical models, and how the completion of the swine genome will promote swine as a biomedical model are discussed.

**Conclusions:**

The completion of the swine genome will enhance the continued use and development of swine as models of human health, syndromes and conditions.

## Background

For several decades swine have been a valuable model for human health and disease [1] and cited in [2]. As a general rule, animal models for human disease provide researchers with the knowledge of disease progression, and insights into new therapies. These potential therapies include drug discovery, validation, and toxicology but also can be extended to include gene therapy, surgical interventions and physical therapy. Rodent models, largely the mouse but more recently the rat, have been used widely to study many human health issues [3]. Unfortunately, as in the case of cystic fibrosis, the rodent models do not always fully mimic the relevant human symptoms.

Genetic modification is often required to create, or improve the quality of, a model. Gene transfer into the genome of livestock species was first achieved in 1985 via pronuclear injection [4]. While this was a tremendous advance, transgenesis by pronuclear injection is limited to adding a gene(s) at a random location, and has the potential to cause insertional mutations [5,6] as well as altering the expression of adjacent host DNA sequences. There have been many attempts to overcome this problem by insertion of large DNA fragments (YAC or BACs) [7], and matrix attachment regions or scaffold attachment regions [8,9]. With the advent of somatic cell nuclear transfer (SCNT) it became possible to select donor cells with the desired integration prior to creating the animal. While SCNT has been useful for transgenesis, the greater advantage is realized when targeting of a gene via homologous recombination is desired. In mice and recently rats, this is accomplished through the use of embryonic stem cells. However, there has been no verified germ-line competent embryonic stem cell in swine. Despite not having a germ-line competent ES cell line, investigators have developed induced pig pluripotent cell lines [10-12] which may prove to be useful for the production of genetically modified pigs. Utilizing SCNT seven genes and 1 transgene have been knocked out in swine: alpha 1, 3 galactosyltransferase (*GGTA1*; [13] the cystic fibrosis transmembrane conductance regulator (*CFTR*; [14,15], Immunoglobulin light chain kappa (IgLK) [16], Immunoglobulin heavy chain (IgH) [17], spinal muscle neuron (*SMN*; [18]), Green Fluorescent Protein transgene (eGFP; [19]), SIGLEC1 (Whitworth et al. 2011 unpublished data) and peroxisome proliferator-activated receptor-gamma (*PPARγ)*; [20], and there are two knock ins [14]). While the efficiency of cloning still remains low [21] a complete swine genome will enhance the identification and modification of genes that are relevant to human disease. This is especially true for genetic modification of existing genes or inserting a transgene at a given location. Prior to completion of the swine genome the first step in developing a construct was to look comparatively at the sequences of other species. The next step was to search both assembled and raw data for similar sequences. And finally the construct could be built. One of the complications that can arise is the presence or absence of multiple members of a gene family. With the latest draft of the genome completed and the limited annotation associated with it we have some assurance that there is only a single copy a given gene, or multiple family members of the gene. A great example here is *SMN*. In humans there are two spinal muscle neuron genes (*SMN1* and *SMN2*). In pigs there is only a single gene for spinal muscle neuron. In humans mutations in both alleles of *SMN1* result in reliance on *SMN2* and the development of spinal muscle atrophy (the number one genetic cause of adolescent mortality in North America). Thus to make a model of spinal muscle atrophy one must not only knockout *SMN*, but also a human *SMN2* must be added as a transgene [18]. Prior knowledge of the number and location of specific sequences makes these projects move forward much more rapidly.

Completion of the swine genome will be a key informational tool for the understanding of human sequences and their potential role in the development of biomedical models. Using comparative genomics, we can begin to investigate and identify cross-species conserved putative genes and regulatory elements as well as single nucleotide polymorphism of genes specific to human diseases. As an example the completion of the human genome, researchers were able to identify the gene mutation that caused sickle cell anemia [22]. Additionally the completion of the swine genome will be useful in the development of new techniques such as cell-based transgensis. Utilizing cell based transgenesis with site specific modification of the pig genome, one can be begin to modify gene function for the enhanced development of novel disease models in several research areas such as cardiovascular disease, xenotransplantation, and neurodegenerative diseases.

### Importance of the pig as a biomedical model

Although the classical model organisms have provided important information about the basic biology of genes and proteins, these models often have limited usefulness due to their inability to sufficiently represent the human disease. In addition, there has been a dramatic decline in the productivity of new drugs [1] which appears to be associated with the current selection of in vivo models. The current animal models do not reflect the pathophysiology of many human diseases well enough to attain sufficient insight into the efficacy of novel drugs, drug therapy or medical devices; e.g. Cystic Fibrosis [14,15,23], Spinal Muscle Atrophy [18] and Parkinson’s Disease [24]. While the pig may not be an obvious choice for a biomedical model, it has been a top choice as a model of human health and disease due to the similarities in anatomy, genetics and pathophysiology.

When compared to other large animal models, swine reach sexual maturity early (6–8 months), have a short gestation length (~4 months) and give birth to multiple offspring. Additionally swine are not seasonal breeders and can therefore produce offspring at any time during the year. Due to the economic and agricultural importance of swine, there is a great deal of information, and logistical support on standardized housing, feeding, and reproductive management and healthcare. Swine also provide a variety of genetic backgrounds as there are numerous breeds of both standard and miniature pigs that have been selected to thrive in a variety of environmental conditions. It may be that the different breeds of swine will represent the various ethnic groups from various regions of the world.

Animal models are essential for insight into etiology and pathogenesis of human diseases and the development of new strategies for disease prevention and treatment. With regard to human anatomy, physiology and pathophysiology, the pig is a favorable animal model [25]. Structure and function of the swine gastrointestinal tract as well as the morphology and pharmacokinetics of the pancreas are similar to humans [26]. However one difference between humans and pigs is the lymphatic system as the cortex and medulla of the swine lymph node is reversed compared to the human lymph node [27]. With similarities and differences between pigs and humans we can begin to utilize genomic tools to analyze human diseases and the molecular mechanisms of these diseases in the pig. Previous genetic analysis of the pig has led to identification of a quantitative trait loci for cutaneous melanoma [28], as well as a novel mutation (Arg to Cys) in the LDL receptor which contributes to spontaneous hypercholesterolemia [29]. Additionally pig models have identified markers for puerperal psychosis [30] and RACK1 as a marker of malignancy for human melanocytic proliferation [31]. As sequences identified in the human are associated with specific disease conditions, an immediate search can be conducted to look for similar genetic variation in the pig. If the variation exists, then those animals can be identified and tested; or if the variation does not exist, then a new genetic modification can be contemplated that would recreate the human phenotype. With a sequenced pig genome and the development of new genetic tools such as SNP chips, investigators will continue to perform genetic analysis of pig populations for molecular mechanisms of human diseases.

### The pig genome

The pig genome has been sequenced and is currently being characterized by the Swine Genome Sequencing Consortium (SGSC) using the hybrid approach combining hierarchical shotgun sequencing of BAC clones and whole genome sequencing [32]. By using the 3x coverage of the BACS and the 3x coverage of the whole genome approach, the SGSC will be able to construct a 6x coverage of the swine genome. Currently the revised assembly (Sscrofa Build 10) has been released and is available for use by the scientific community (ftp://ftp.ncbi.nih.gov/genbank/genomes/Eukaryotes/vertebrates_mammals/Sus_scrofa/Sscrofa10.2/ or http://www.ncbi.nlm.nih.gov/genome/guide/pig/).

The swine genome is comprised of 18 autosomes and 2 sex chromosomes (X and Y chromosomes) and is estimated to be 2.7 Gb. The pig genome is ~7% smaller than the human, while the mouse and dog genomes are 14% smaller. In addition to the similarity in size of genome, there is extensive homology of the swine genome to the human. On a nucleotide level swine are 3x more similar to humans than mice are to humans [34]. On average the synteny blocks of swine-human are farther down the phylogenetic tree than the mouse or dog [34]. Since there are larger syntenic blocks between swine and humans, positional cloning from the pig is generally straightforward and local regulatory interactions between enhancer regions are more likely conserved.

## Methods

### Current technologies to produce a TG pig

**Figure 1:**
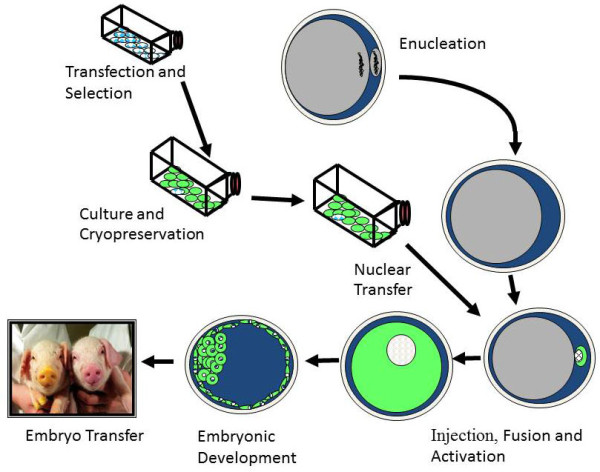
**The Somatic Cell Nuclear transfer procedure used to create new genetic modifications [**[35]**].**

While generation of germ-line modified swine via embryonic stem/germ cell technology has not been reported a number of other technologies can be used. These include pronuclear injection [4], oocyte transduction [34,35], embryo transduction [36,37] fibroblast transduction followed by SCNT [35], sperm-mediated gene transfer (SMGT) [38], fibroblast transfection followed by SCNT [34,35], and embryonic germ cell transfection followed by NT [39]. The main advantage of pronuclear injection is that large constructs can be integrated. The main disadvantage of pronuclear injection, oocyte transduction and SMGT is the lack of control over the site of integration. The disadvantage of SCNT is that cloning sometimes results in abnormal animals [13,40] and the efficiencies of producing offspring is low. In addition, porcine fetal fibroblasts used as SCNT donor cells have a finite proliferation capacity and a lower gene targeting frequency as compared to embryonic stem cells available in other species [41]. Despite these challenges, cloning from a modified somatic cell is the only method (Figure 1) available for the production of targeted modifications in swine, and these modifications can be determined in cells prior to making the animal [42].

Potential solutions for improving cloning efficiency have recently focused on the epigenetic regulation of development. Completion of the swine genome will be instrumental in these efforts. Successful cloning by SCNT depends on erasure of somatic cell epigenetic modifications (e.g. histone acetylation and genomic DNA methylation) during pre-zygotic reprogramming, followed by a post-zygotic establishment of embryonic modifications that regulate transcription [43,44]. This reprogramming is critical to the normal development of cloned animals. Chemical treatments to alter DNA methylation and histone modifications in SCNT donor cells and embryos are used to shift the genome epigenetic state to more closely resemble that of a normally fertilized zygote. This “assisted remodeling” includes the addition of histone deacetylase inhibitors (HDACi) to donor cell and embryo culture medium. One example, Trichostatin A (TSA), can improve in vitro development of SCNT embryos, but TSA is a known teratogen, and adverse effects on development and survival have been reported in some species [45]. Other HDACi with lower toxicity such as Scriptaid [45,46] and sodium butyrate (NaBu; [47]) can markedly improve pig SCNT embryo development when used to treat donor cells and embryos, respectively. To mimic erasure of DNA methylation in donor cells or reconstructed embryos, 5-aza-20-deoxycytidine (5-aza-dC) treatment has been used on bovine donor cells prior to SCNT [48]. This treatment alters the characteristics of the donor cells but does not enhance development in vitro. In swine, 5-aza-dC applied to IVF-produced pig zygotes reveals that DNA of early pig embryos is not subject to active demethylation at the pronuclear stage or to passive demethylation during the cleavage stages [49], as occurs in the mouse [44]. Such differences in response to chemical treatment among species and stage of development highlight the variable nature of the cloning procedure on epigenetic gene regulation (imprinted or non-imprinted). With the more recent DNA sequencing technologies that produce millions of reads there is increasing need for a scaffold to which these reads can be aligned. If these reads were generated by chromatin immuno-precipitation, e.g. methylated DNA, or histones with specific post-translational modifications, then their alignment to a scaffold that has predicted CpG islands and/or transcription initiation sites would provide insight to regulation of the development of the cloned (or normal) embryo. It may also explain the results from experiments that evaluate the transcriptional profile after, for example, HDACi treatment [50]. Knowledge of the complete pig genome sequence will provide a more detailed understanding of this epigenetic regulation at specific regulatory regions of genes and enable swine-specific refinement of SCNT treatments to improve cloning outcomes [21].

## Results

### Genetically engineered biomedical models

Arguably, the first genetically engineered pigs that modeled a human disorder were produced as an unintended consequence of an attempt to modify growth in production pigs [4]. The growth hormone gene was linked to the methalothionine promoter in the hope of regulating growth by alterations of zinc in the diet. Although the strategy did provide a degree of regulation of the transgene, the basal expression of growth hormone in the uninduced state was sufficiently high to produce an acromegaly phenotype with associated arthritis symptoms. Although some researchers suggested a value of these pigs as a model [51], the transgenic pigs primarily served to help guide future efforts to manipulate growth in agricultural settings.

In 1996, the first intentional transgenic pig model of a human disorder was described for retinitis pigmentosa (RP) [52]. In this genetic disorder, there is a loss of rod cells around puberty that results in loss of sensitive vision (“night blindness”). The loss of rods is followed by the progressive loss of cone cells resulting in loss of peripheral vision that eventually progresses to complete blindness. When transgenic mice were made with the mutant gene, they too developed RP [53]. However, researchers wanted to develop therapeutic interventions with the goal of maintaining the cone cell population after the condition has been diagnosed. The mouse retina, with very few cones, does not provide an adequate model for this phenomenon. However, the pig has a rod to cone ratio almost identical to humans. This model and similar models continues to be used today.

Transgenic pigs will continue to help us to understand the role of genes, advance pharmacological pharming and develop organs for xenotransplantation. In addition, these models will help us to study disease and establish safety and efficacy of new drugs and procedures. Genetic engineering in swine presents the opportunity for targeted genetic manipulations. For an exhaustive list of genetic modifications reported in swine see a review by Whyte and Prather [54]. Below are a few examples of where genetically modified swine have provided insight not otherwise obtainable.

### Cystic fibrosis pigs

In 1938, cystic fibrosis (CF) of the pancreas was first described [55]. Since that time, it has been learned that many other organs are involved (lung, intestine, liver, sweat gland, gallbladder and male genital tract) [56]. Cystic Fibrosis is an autosomal recessive disease where approximately 5% of Caucasians are carriers [57]. Despite identification of the cystic fibrosis transmembrane conductance regulator (CFTR) anion channel as the causative gene for CF and having a mouse model where *CFTR* has been disrupted [58], for over 20 years, little advancement has been made in the understanding of CF. Unfortunately, disruption of *CFTR* in the mouse does not result in a human CF phenotype [59-61]. For those symptoms that are presented in mouse models, such as the intestinal obstruction phenotype that is observed in *CFTR*−/− mice, the presentation differs from what is observed in humans. In 2008, Rogers et al. reported the development of *CFTR*−/− and *CFTR ΔF508* swine. Swine were chosen because of their similarity to humans in their anatomy, biochemistry, life span, and genetics [56]. Homologous recombination was used to disrupt *CFTR* in outbred porcine fibroblast cells. This followed by SCNT and subsequent matings produced homozygous *CFTR*−/− and *CFTR ΔF508/ΔF508* animals. In approximately 15% of human CF patients there is an intestinal obstruction observed in the first 48 hours after birth, while in the pig 100% of the animals are affected [14,15]. In addition to the intestinal phenotype these pigs further recapitulate the human phenotype by display of a blocked bile duct, liver lesions, blocked pancreatic duct, and a congealed gallbladder [15,62]. These CF pigs also develop the hallmark phenotype of lung disease [23] and have led to a better understanding of the underlying causes of CF in humans. Unexpectedly, the pig has also led to the identification of low levels of IGF-1 as a possible cause of smaller than average stature among CF patients [63]. The CF pigs will continue to provide the necessary understanding for development of drug therapies or treatment that will aid in prevention of the disease. Prior knowledge from a sequenced genome that there were not multiple gene family members of CFTR would have reduced the risk of the overall project.

### Xenotransplantation

Xenotransplantation is the transplant of cells, tissues or organs from one species to another. The number of people waiting for a suitable organ is over 108,000 (http://www.unos.org/) and there are probably that many people that could benefit from an organ, but are not ill enough to get put on the waiting list. Approximately 10 new patients a day are added to the UNOS waiting list (http://www.unos.org). The number of organ donors for 2009 was only ~14,000. One way to meet the demand by patients suffering from a wide variety of chronic diseases and end-stage organ failure may be by xenotransplantation. Swine organs may be able to satisfy the unmet and clinical need, but preexisting antibodies that recognize an alpha-1-3-galactosyl (α-gal) epitope result in hyperacute rejection (HAR; [64]). Within minutes swine cells or organs transferred to non-human primates, such as baboons, are destroyed. The α-gal residues are synthesized by an enzyme, alpha-1,3-galactosyltransferase, which is encoded by *GGTA1.* GGTA1 is a pseudogene in humans, apes, and Old World monkeys; however, *GGTA1* is functional in most mammals including the pig. In order to utilize swine as a model for xenotransplantation, one of the first obstacles was to remove the α-gal epitopes on the swine cells by disruption of *GGTA1*[13,65-72]. Other strategies employed to help avoid HAR are the addition of *CD55*, and/or *CD59* on the *GGTA1* background, or addition of transgenes encoding enzymes to create carbohydrate structures to cover the gal epitope [73]. In addition to HAR, other genetic modifications have been made to address cell-mediated, acute humoral xenograft, and non-vascular rejections as well as the potential cross species transmission of porcine endogenous retroviruses (PERV; [73]). PERVs are an integral component of the swine genome which are ubiquitously expressed and have has many as 50 proviral loci in the genome depending on the breed of pigs [74]. The majority of the PERVs are defective and are not disease causing in the pigs [75,76]. There are however currently three replication competent subclasses of PERVs (PERV A, B, & C) that can infect either human (PERV A and C) or pig cells (PERV B) in vitro. With these three subclasses of PERVs that can potentially infect human cells there is a risk of cross species transmission during xenotranplantation. Concerns of PERV infection during xenotransplantation range from acceleration of rejection of the xenograft through a T-cell mediated rejection to providing the necessary sequence to convert an endogenous retrovirus to a replication competent retrovirus [74].

With genetic modifications short term solid organ xenotransplantation from pig to non-human primate has been achieved with 2–6 month survival of heterotropic heart transplants and with life supporting kidney transplant for 3 months [77]. However, lung and liver transplants have not been as successful as the heart and kidney due to thrombotic microangiopathy and coagulation dysfunction. The next big hurdle for xenotransplantation is the acute vascular rejection (AVR) that occurs within hours to days after the transplant. AVR is usually characterized by endothelial activation and cellular damage from thrombotic microangiopathy [78]. However AVR may be overcome by the utilization of genetic engineering. Oropeza et al., [78] reported that the expression of human A20 gene (a TNF-alpha inducible factor) in pigs can provide the protection against apoptotic and inflammatory stimuli. The ability to prolong survival of the pig xenograft appears to be modulated on the ability to produce multi-transgenic pigs with sufficient expression levels of all genes involved. The completed genome provides information about the presence and similarity of many of these human genes in the pig. If a given gene product is predicted to be highly homologous to the human protein, then it may not be necessary to add or disrupt that gene for successful xenotransplantation.

### Diabetes

Diabetes mellitus is a group of metabolic disorders characterized by hyperglycemia resulting from impaired insulin secretion or insulin action or a combination of both. In the physiologic state, the two incretin hormones glucose-dependent insulinotropic polypeptide (GIP) and glucagon-like peptide-1 (GLP-1) enhance insulin secretion in a glucose-dependent manner. GIP and GLP-1 are secreted from enteroendocrine cells into the blood in response to nutrients and bind to their specific G-protein coupled receptors, GIPR and GLP-1R respectively, on the pancreatic β-cells. In type 2 diabetic patients the incretin effect is highly reduced which is mainly related to an impaired insulinotropic action of GIP while the insulinotopic action of GLP-1 is preserved [80]. In order to evaluate the consequences of an impaired GIP action on glucose control and pancreatic islet integrity, transgenic pigs expressing a dominant-negative GIPR (GIPR^dn^) in the pancreatic islets were generated [80] by lentiviral gene transfer [36].

*GIPR*^*dn*^ transgenic pigs resemble characteristic features of human type 2 diabetic patients with impaired insulinotropic action of GIP, reduced glucose tolerance and insulin secretion as well as a reduction of β-cell mass, and are therefore a relevant model for numerous applications in basic as well as in translational research. One area of translational research is the development and preclinical evaluation of incretin-based therapeutics, which is a very active field of clinical research [81,82]. These include GLP-1 receptor agonists (GLP-1 analogues and GLP-1 mimetics) as well as inhibitors of the enzyme dipeptidyl peptidase-4 (DPP-4) [83], which rapidly degrade incretin hormones in vivo. Again, a completed genome will provide the number of family members and similarity of the members of the glucose homeostasis regulatory pathway(s). Such knowledge will better direct research to create better models, treatments and therapies for diseases such as diabetes.

## Discussion

### Genetic engineering and genetic tools

Introduction of exogenous DNA into the swine fibroblast prior to SCNT or into the pronucleus or early embryo [84-88] has been performed utilizing several different strategies which have included lipid based delivery [89,90], viral delivery [13,14,57] and electroporation [65,70,92]. Even though these methods can produce genetically modified swine, these methods utilize random integration of the exogenous DNA into the swine genome that occurs utilizing the double strand DNA break repair system. Since DNA integration employs the double stranded DNA break repair system, linearized DNA will integrate 5x greater than supercoiled DNA [92]. One of the pitfalls of this breakage of plasmid DNA is that cells could potentially be drug-selected positive but not express the transgene. In addition, it is thought that concatamers are more likely to occur with the random integration of plasmid DNA which also can potentially silence the transgene [93,94]. Concatamers are head to tail multicopy gene arrays of the exogenous DNA that can occur before or during integration [92]. Another drawback to random integration of exogenous DNA is potential for insertional mutations of the host genome that may alter the phenotype of the organism [5,6].

Gene targeting is a more precise event than pronuclear injection as it utilizes specific modifications to the genomic sequence. Gene modification or introduction of transgenic constructs into the genome of pig donor cells commonly relies on homologous recombination (HR) as described originally in mouse ES cells [95-97]. These HR events can be used to insert large fragments of DNA; however, to be successful >3 kb of homologous DNA needs to be used in the targeting vector. Porcine fibroblasts can be transfected with a targeting vector containing the desired mutation. In a small number of cells the targeting vector pairs with the analogous chromosomal sequence, introducing the mutation to the genome by homologous recombination. Cells, screened and identified as targeted, are then isolated and maintained as a clonal population. Targeting frequency by HR is only one in 10^5^-10^6^ cells, even with tissue culture selection procedures [98].

Gene targeting is a valuable tool for the study of *in vivo* gene expression however sometimes these targeted events can be embryonic lethal. There are genetic methods to avoid this embryonic lethality but still investigate the gene of interest such as inducible and conditional gene inactivation in a tissue specific and time specific manner. The Cre/loxP recombinase system is the most widely used conditional system [99]. The Cre/lox P system utilizes Cre a 38 kDA recombinase from bacteriophage P1 and loxP which is a 34 bp consensus sequence. The use of lox P sites in the same orientation will excise any sequence between them when an active Cre is present [99]. The Cre-lox system allows for time specific expression of your transgene in a tissue specific manner if necessary. The Cre-recombinase system has been confirmed to be functional in swine cells under physiological conditions (Wells, unpublished).

Knowledge of the sequence of the gene of interest, as well as the alternative splice sites, copy number of the number of members of a gene family are very important in developing genetic modifications as described above.

#### Cell based transgenesis

With the sequenced pig genome, the use of cell based transgenesis will become a new approach to make genetically modified pigs. Cell based transgenesis allows investigators to make more precise genetic modification by utilizing natural occurring events within the genome such introduction of double stranded DNA breaks at targeted sites.

**Figure 2:**
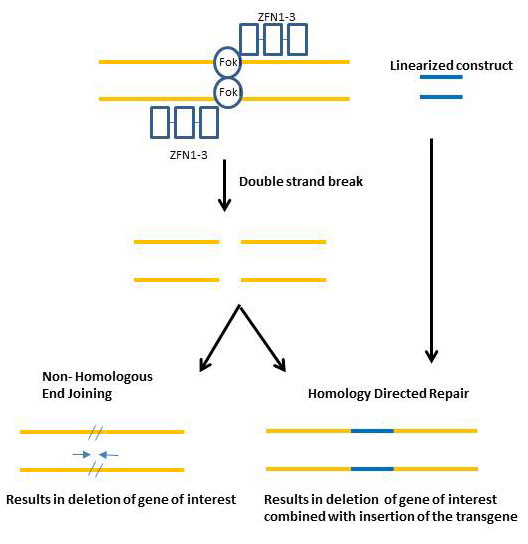
**A series of three Zinc-Finger nuclease (ZFN 1–3) bound to FokI cleavage domain to form a dimer at a sequence specific site to introduce a double stranded break.** The double stranded break introduced by the ZFNs will either repair itself with the Non-homologous end joining mechanism which results in the deletion of base pairs (1–300 bp) from the gene of interest. Alternatively a linearized construct can be introduced with the ZFNs and the repair will use the homology directed repair which inserts the transgene.

Zinc fingers nucleases (ZFNs) [19,100] have been used recently to target genetic modifications with a high efficiency. ZFNs have been used in mammalian cells to increase the rate and specificity of gene alteration and transgenesis. ZFNs are synthetic proteins composed of a nonspecific *Fok*I cleavage domain and multiple Cys_2_His_2_ zinc finger DNA-binding domains (Figure 1) [101]. The binding of two ZFN-*Fok*I heterodimers to two target sequences on the coding and non-coding DNA strands with a 6 bp separation allows *Fok*I dimerization and subsequent DNA cleavage. The resulting double strand break can increase the frequency of HR-mediated gene targeting by approximately 1000-fold (reviewed in [102]). Linking zinc fingers in tandem to form modules allows for the design of highly specific DNA recognition sequences [103]. The increase in gene targeting by ZFNs is achieved by the activation of DNA repair mechanisms. If transgene DNA is co-transfected with ZFNs, repair of the double strand breaks by HR can result in transgene insertion in close proximity to the cleavage site as shown in Figure 2[104]. In the absence of donor DNA, repair of the ZFN cut-site by non-homologous end joining (NHEJ) can inactivate a gene by generating localized insertions or deletions (Figure 2) [105]. Recently, commercially produced ZFNs (Sigma-Aldrich, Inc. St. Louis, MO) were used to disrupt a GFP transgene in a primary culture of porcine fetal fibroblasts [100] which were then used to create live piglets [19]. In addition PPARγ has been knocked out by the use of ZFN technology [20]. Insertion of transgenes and/or gene deletions generated by ZFNs in pig fibroblasts could potentially enhance the efficiency of transgenic swine by SCNT. Currently there is a large undertaking of knocking- out 100 genes related to cardiovascular and renal disease in the rat genome using ZFNs or TALENs (Geurts 2011 personal communication).

Recently, there have been several reports of using Transposons for the production of genetically modified organisms such as fish, frogs, mice, rats, and swine [106-112]. Transposons are discrete and mobile sequences in the genome that do not rely on relationships with other sequences. In mammalian transgenesis, two transposons *Sleeping Beauty* and *piggybac* have been used for production of genetically modified swine [106,107,113,114]. The transposon system works by having the transgene of interest flanked on both sides with specific terminal repeats that will be used as transposase recognition sites. Once in the host cell, transposase will insert the transgene of interest into the host genome. Modifications to this system have been made to increase the efficiency of the transposon system. Artificial methylation of the transposon, and preference for the plasmid to be in the supercoiled conformation results in an increase number of founders that express the transgene and nearly eliminated the insertion of concatemers into the host genome [115].

## Conclusions

The development of genetically engineered pigs for human health and disease is having a significant impact on the scientific community as well as improving the development of treatments and therapies for human diseases. Currently genetically engineered pig models are being used for analysis of gene function in various human diseases, development of new therapeutic strategies as well as production of biopharmaceutical products. For example it is estimated that 60–100 transgenic pigs could produce enough Factor IX needed for all the hemophilia B patients in the United States (Velander 2011 personal communication). Presently, there are 7 genes and 1 transgene that have been knocked out/in swine and more are being added to this list each year. Genetically engineered swine modified for specific diseases will permit the invasive monitoring of the development of diseases that previously were beyond the grasp of physicians who only saw the disease in humans. The tools are now available to recreate in swine most any genetic disease that occurs in humans. These new swine models can then be used to test interventions pre-clinical and thus reduce any risk for patients. The completion of the swine genome is providing the platform for discovering which genes are responsible for various genetic diseases, as well as the tool for recreating these mutations in swine such as the increased use of cell based transgenesis in a site specific manner.

## Competing interests

The authors declare that they have no competing interests.

## Authors’ contributions

This review is an International collaboration between the authors promoting the pig as a large animal biomedical model for the scientific community. Many of these experiments were conducted in the various laboratories of the authors. All authors have read and approved the final manuscript.

## Pre-publication history

The pre-publication history for this paper can be accessed here:

http://www.biomedcentral.com/1755-8794/5/55/prepub
